# Machine Learning Approach to Predicting COVID-19 Disease Severity Based on Clinical Blood Test Data: Statistical Analysis and Model Development

**DOI:** 10.2196/25884

**Published:** 2021-04-13

**Authors:** Sakifa Aktar, Md Martuza Ahamad, Md Rashed-Al-Mahfuz, AKM Azad, Shahadat Uddin, AHM Kamal, Salem A Alyami, Ping-I Lin, Sheikh Mohammed Shariful Islam, Julian MW Quinn, Valsamma Eapen, Mohammad Ali Moni

**Affiliations:** 1 Department of Computer Science and Engineering Bangabandhu Sheikh Mujibur Rahman Science & Technology University Gopalganj Bangladesh; 2 Department of Computer Science and Engineering University of Rajshahi Rajshahi Bangladesh; 3 iThree Institute Faculty of Science University Technology of Sydney Sydney Australia; 4 Complex Systems Research Group Faculty of Engineering The University of Sydney, Darlington Sydney Australia; 5 Department of Computer Science and Engineering Jatiya Kabi Kazi Nazrul Islam University Mymensingh Bangladesh; 6 Department of Mathematics and Statistics Faculty of Science Imam Mohammad Ibn Saud Islamic University Riyadh Saudi Arabia; 7 School of Psychiatry Faculty of Medicine University of New South Wales Sydney Australia; 8 Institute for Physical Activity and Nutrition Faculty of Health Deakin University Victoria Australia; 9 Healthy Ageing Theme The Garvan Institute of Medical Research Darlington Australia; 10 WHO Collaborating Centre on eHealth, UNSW Digital Health School of Public Health and Community Medicine, Faculty of Medicine University of New South Wales Sydney Australia

**Keywords:** COVID-19, blood samples, machine learning, statistical analysis, prediction, severity, mortality, morbidity, risk, blood, testing, outcome, data set

## Abstract

**Background:**

Accurate prediction of the disease severity of patients with COVID-19 would greatly improve care delivery and resource allocation and thereby reduce mortality risks, especially in less developed countries. Many patient-related factors, such as pre-existing comorbidities, affect disease severity and can be used to aid this prediction.

**Objective:**

Because rapid automated profiling of peripheral blood samples is widely available, we aimed to investigate how data from the peripheral blood of patients with COVID-19 can be used to predict clinical outcomes.

**Methods:**

We investigated clinical data sets of patients with COVID-19 with known outcomes by combining statistical comparison and correlation methods with machine learning algorithms; the latter included decision tree, random forest, variants of gradient boosting machine, support vector machine, k-nearest neighbor, and deep learning methods.

**Results:**

Our work revealed that several clinical parameters that are measurable in blood samples are factors that can discriminate between healthy people and COVID-19–positive patients, and we showed the value of these parameters in predicting later severity of COVID-19 symptoms. We developed a number of analytical methods that showed accuracy and precision scores >90% for disease severity prediction.

**Conclusions:**

We developed methodologies to analyze routine patient clinical data that enable more accurate prediction of COVID-19 patient outcomes. With this approach, data from standard hospital laboratory analyses of patient blood could be used to identify patients with COVID-19 who are at high risk of mortality, thus enabling optimization of hospital facilities for COVID-19 treatment.

## Introduction

SARS-CoV-2 has caused the current pandemic of COVID-19, a disease that first emerged as an outbreak in December 2019 in the Chinese province of Hubei [1]. The management of patients with COVID-19 remains problematic and controversial, although this is to be expected in such a recently emerged disease. The first symptoms of COVID-19 resemble those of many other infections and inflammatory conditions that affect the respiratory system; they include fever, sneezing and rhinitis, persistent cough, and fatigue with body ache [2]. However, an infected patient can rapidly develop additional and more severe symptoms that can be life-threatening and require intensive care intervention; these include pneumonia, severe shortness of breath, diarrhea, dispersed thrombosis, and vascular inflammation [3,4]. An additional issue in caring for patients with COVID-19 is the presence of comorbidities that interact with COVID-19, particularly pulmonary and vascular conditions, which can greatly worsen the patient’s prognosis [5]. This is an important consideration given the current lack of effective therapy for COVID-19. However, there have been notable advances in treating patients with advanced disease; therefore, the ability to predict that a patient will have poor outcomes, indicating a need for more aggressive treatment, has the potential to save lives and enable more effective allocation of resources.

Intensive care units (ICUs) are key to increasing the survival of patients with severe COVID-19; they provide oxygen, 24-hour monitoring and care, and assisted ventilation when needed. Therefore, ICU beds are a precious resource in locations where COVID-19 case numbers are high [6-8]. Allocating hospital wards or ICU beds for infected patients thus requires rapid decision-making processes, both to use resources efficiently and reduce patient suffering and mortality. In many parts of the world, stressed care systems face significant difficulty in deciding on ICU bed allocation; therefore, a smart, automated system could be useful to improve care and resource allocation. The World Health Organization has recommended that all suspected patients with COVID-19 be tested by reverse transcription–polymerase chain reaction (RT-PCR)–based diagnosis methods that directly detect viral RNA [9]. Testing by approaches other than RT-PCR does not yet show acceptable accuracy. However, RT-PCR tests can take many hours or days to finalize the test outcomes, by which time the health condition and infectious status of confirmed patients may deteriorate. Rather than seeking a new single rapid test that improves on RT-PCR, an alternative approach could be to use results from many different profiling tests that are already available and can be performed quickly using existing equipment [10,11]. The best way to use the resulting multidimensional data is currently controversial.

Rapid blood and serology testing of clinical samples by current equipment enables monitoring of many peripheral blood parameters of interest, some of which indicate changes in organ functions and are used to diagnose a range of conditions and diseases [7,12]. This raises the possibility that such profiling of blood samples could provide predictive information about the disease trajectory and risk of comorbidities for patients with COVID-19. Some data is already used in physician deliberations; however, the many available test parameters suggest that an agnostic statistical or machine learning (ML) approach would improve the quality of those decisions. Therefore, we undertook a comprehensive assessment that examined the utility of a range of statistical and ML approaches. Indeed, we identified algorithms that showed significantly improved outcome estimates. Therefore, this work has the potential to optimize decision processes regarding patient care by clinicians who are under significant time and resource pressure during the current COVID-19 pandemic.

## Methods

### Data Sets and Analyses

**Figure 1:**
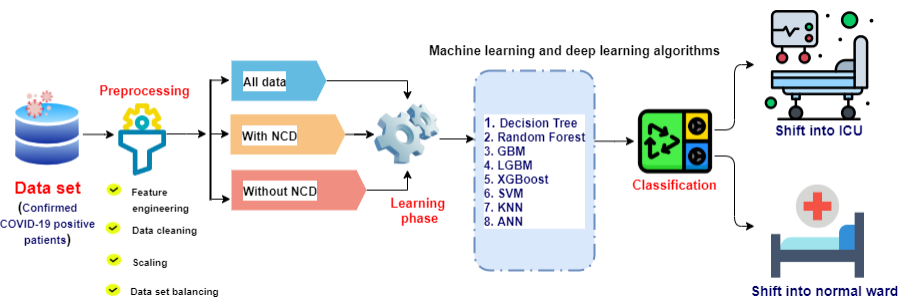
Proposed methodology and workflow of the machine learning analysis in this study. ANN: artificial neural network; GBM: gradient boosting machine; ICU: intensive care unit; LGBM: light gradient boosting machine; NCD: noncommunicable disease; SVM: support vector machine; KNN: k-nearest neighbor; XGBoost: extreme gradient boosting.

We used two different data sets in this study; the first included data from 89 patients, and the second included data from 1945 patients with confirmed positive COVID-19 tests identified by RT-PCR. For the first data set [13], we use statistical methods such as the Student *t* test, chi-square test, and Pearson correlation to identify the most significant and associative blood parameters that can strongly distinguish between patients with COVID-19 and healthy people. Moreover, to compare the blood parameter values of patients with COVID-19 with those of healthy patients, we considered the standard value ranges as reference values for each parameter. For the second data set [14], in addition to statistical methods, we used several ML models to further identify blood parameters that can discriminate between COVID-19–positive patients who are at risk of serious illness and those who are not. Figure 1 depicts a schematic of the ML analysis workflow of our approach.

We formulated the task of identifying patients with severe COVID-19 to enable selection of the appropriate hospital ward for their care as a classification problem by training ML models with features of clinical data collected from blood samples of patients with COVID-19. Raw data of interest collected from the data sets underwent a data-wrangling pipeline, including denoising, missing value imputation, transformation, normalization, and partition. Next, several statistical comparisons and correlation methods were adopted for feature engineering, including the Student *t* test, chi-square test, and Pearson correlation. After this, each data set was further split into three categories based on the criteria of existing noncommunicable disease (NCD): with NCD, without NCD, and all data. In our study, “NCD” refers to patients with pre-existing noncommunicable diseases or conditions. Finally, a range of state-of-the-art ML methods were trained and evaluated. The algorithms used included decision tree (DT), random forest (RF), gradient boosting machine (GBM), extreme gradient boosting (XGBoost), support vector machine (SVM), light gradient boosting machine (LGBM), k-nearest neighbor (KNN), and artificial neural network (ANN)–based deep learning sequential models. Each of these steps is discussed in the following subsections.

### Data Collection

We obtained two different data sets of patients with COVID-19. The first data set was produced by Zenodo [13], and it contains demographic information and blood sample information from 89 COVID-19–positive patients. In this data set, 31 patients were alive at the point of data collection, while 58 patients had died. The second, larger data set was obtained from the Kaggle web-based resource [14], which contains grouped information regarding previous diseases, blood sample results, and vital sign data of 1945 COVID-19–positive patients. The primary sources of the data in this set are Brazilian hospitals, including Sirio Libanes, São Paulo, and Brasilia. The parameters of the data set included patient age percentile, gender, and demographic information. Some patients had pre-existing NCDs, including hypertension and immunocompromised status. The blood parameters examined included lactate, respiratory rate, diastolic blood pressure, hemoglobin, hematocrit, venous base excess, leukocytes, neutrophils, albumin, arterial base excess, urea, platelets, potassium, systolic blood pressure, venous PO_2_, arterial O_2_ saturation, partial thromboplastin time, temperature, gamma-glutamyl transferase, venous O_2_ saturation, creatinine, international normalized ratio (INR), venous PCO_2_, venous pH, arterial bicarbonate, labels of free fatty acids, venous bicarbonate, calcium, lymphocytes, alanine aminotransferase, aspartate aminotransferase, arterial PCO_2_, dimerized plasmin fragment D (D-dimer), oxygen saturation, bilirubin, arterial PO_2_, arterial pH, heart rate, blast, and glucose. During the feature-engineering phase in our study, all these blood parameters were considered as features.

### Data Processing

For the Zenodo data set [13], which consists of 89 COVID-19–positive patients, we first removed any unwanted parameters (eg, ethnicity, BMI, drinking or smoking habits). We then eliminated all the missing values, resulting in a data set of 70 patients. In the Sirio Libanes data set [14] from Kaggle, there were 1945 individual patients with 54 types of tests. The primary data set contained a large number of missing values. This data set was prepared from information received from local hospitals and some of this information was not well prepared, which is a significant reason why most of the data have missing entries. The rationale behind the removal of entries with missing parameter values is that when we conducted a pilot study with the imputation of missing values with mean, median, or regression values, poor predictive performance was observed. In the raw data set, the dimensions were 1925 × 205, and almost 57% of the data units (cell values) were missing; after eliminating unwanted attributes, the amount of missing data increased above 70%. If we considered all the data and imputed the missing values, most of the values would be inferred, and the analysis results would be unreliable. Therefore, we eliminated entries that contained at least one missing value. This elimination resulted in 545 sets of patient data entries in the second data set that contained no missing values. Among the patients in this data set, 264 had sufficiently severe symptoms to be admitted to the ICU. Both data sets underwent a denoising step, in which we removed unwanted strings. Standard scaling techniques were performed, such as feature scaling, in which the variance values of the data are scaled between 0 and 1; this is calculated by subtracting the mean value of a feature from the original value and then dividing by the standard deviation. After preprocessing, we considered data from 545 patients for the analysis. For a precise study, we then divided this data set according to whether a patient had a coexisting NCD (NCD) or not (no NCD). We found 264 patients with NCDs and 281 patients without NCDs; in the NCD and no NCD groups, 156 and 108 patients were respectively classed as displaying severe conditions. After this data preparation and preprocessing, we considered all these data for the statistical analysis. Due to the possibility of data leakage in ML analysis if we separated the test set and train sets after preprocessing, we first separated a randomly selected 80% of the grouped patient data for model training and used the rest for model validation testing, then performed the preprocessing steps.

### Statistical Methods to Identify the Most Significant and Associative Blood Parameters

In the statistical analysis, we used chi-square tests for categorical variables, Student *t* tests for continuous variables, and Pearson correlations among various blood sample counts. The null hypothesis was that the data from the patients with COVID-19 and the healthy population were independent. Significant blood parameters were chosen based on a *P* value <.05, while in some cases, the selection criteria were a false discovery rate–adjusted *P* value <.05 and an absolute value log 2 fold change (LFC) <1. To understand the changes (positive or negative) of the parameters and the number of changes, we have calculated the LFC. LFC=1 indicates a fold change of value 2. Furthermore, hierarchical clustering was conducted on the Pearson correlation coefficients for grouping significant parameters [15-17].

### ML Models to Classify COVID-19 Disease Severity

To identify a set of important blood samples as a feature selection step, we employed a set of ML algorithms using COVID-19 data sets that included data from severely and nonseverely affected patients. We chose ML algorithms that are known to perform classification tasks with superior performance and fast execution [18,19]. For this purpose, we considered a basic ensemble learning approach based on max-voting, averaging, and weighted averaging for some classifiers, as well as advanced ensemble learning algorithms that function by stacking, blending, bagging, and boosting. Ensemble learning algorithms are combinations of one or more basic algorithms that are high-performing, efficient, effective, and easy to debug [20,21].

We next address the parameters of the ML algorithms that were considered when they were run. In the DT algorithm, we used a random state of 42, a criterion of Gini, and a minimum sample split of 2. Similarly, in the RF algorithm, the minimum sample split was 2 and the number of estimators was 100. Degree and kernel cache size are parameters of the SVM algorithm; the algorithm sets a polynomial kernel with a degree of 3, and we set the kernel cache size at 200 MB for fast execution. In the GBM algorithm, the learning rate was 0.1, the criterion was friedman_mse, and the number of estimators was 100. The learning rate in the LGBM algorithm was 0.05, the feature fraction was 0.9, the bagging fraction was 0.8, and the bagging frequency was 5. In the XGB algorithm, we used a tree-based booster with a maximum depth of 6, a learning rate of 0.1, and 1000 estimators. For the KNN algorithms, we used Minkowski matrices; the weights were uniform, and the number of neighbors was 3 (k=3).

We also experimented with a sequential deep learning model, namely, a feed-forward 1D ANN. This model consists of an input layer, three hidden layers, and an output layer [22]. Each layer contains a collection of parallel processing nodes, called neurons, that take input from the nodes of the previous layer. All the hidden layers are activated by rectified linear units, and the output layer is activated by a softmax function, providing the class probability of the input sample. The network was trained in 1000 epochs using the stochastic gradient descent optimization algorithm with categorical cross-entropy loss as a convergence indicator and a learning rate of 0.0001.

### Shapley Additive Explanation Value Calculations

To measure the feature importance, we calculated the Shapley Additive Explanation (SHAP) values from all the models to estimate the degree of contribution of each of the features in the samples of the training data set to the overall decision-making of the model [23]. SHAP uses game theory rules to determine the contributions of particular features to the decision-making of the model. We used the TreeExplainer [24] for tree-based models and the KernelExplainer [23] for kernel-based models to calculate the feature importance. After finding the SHAP values for all the models, we normalized the values in a fixed range and considered the average values.

### Evaluation Matrices for the ML Models

We evaluated the performance of our models using precision, recall, F1 score, the area under the receiver operator characteristic curve (AUC-ROC), and the log loss function. The precision depicts the proportion of true positive instances among all the predicted positive instances [25]; in contrast, the recall shows the proportion of the actual true instances that are predicted positively by the models [25]. The F1 score is the harmonic mean of precision and recall [25]; we calculated the F1 scores to achieve better evaluation between precision and recall. The AUC of a classifier is equivalent to the likelihood that the classifier will rank a randomly selected positive value higher than a randomly selected negative value [26]. Log loss is also essentially used as a metric for classification; it is calculated by the probability of actual and predicted classes [27]. Log loss is among the most useful evaluation metrics. The function can be described as below:


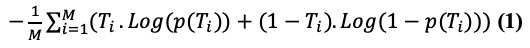


where M depicts the number of classes, T_i_ indicates the actual class, and p(T_i_) indicates the probability of that class.

## Results

### Analysis Approaches

In this study, we adopted two scenarios for analyzing research data. In the first scenario, we applied the Student *t* test and Pearson correlation to the blood cell parameters of COVID-19–positive patients and the normal ranges of the blood cell parameters. We found that both statistical approaches yielded predictive capability of immature granulocytes (absolute), hemoglobin A_1c_, fibrinogen, and lipase as significant for COVID-19–positive patients. In the second scenario, we accounted only for COVID-19–positive patients in the severity calculation. We also applied two different analysis approaches. The first one was the Student *t* test, and the second was a set of ML methods. Using both of these approaches, we found that respiratory rate, lactate, blood pressure (systolic and diastolic), hemoglobin, hematocrit, venous and arterial base excess, neutrophils, albumin, urea, platelet count, and potassium were good indicators of the patients’ disease severity and represented a small set of predictors of COVID-19 severity measurements.

### Patient Demographics

**Figure 2:**
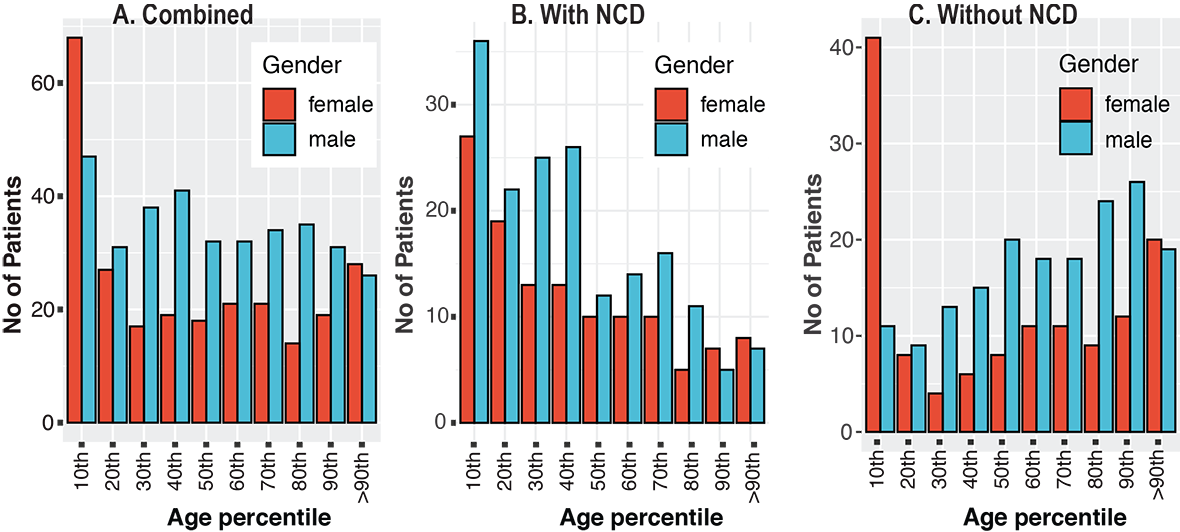
Age percentiles of patients with COVID-19 for (A) both patient groups, (B) patients with NCDs, and (C) patients without NCDs. NCD: noncommunicable disease.

A comparison of the demographic information for the data from the patients with severe and nonsevere symptoms is shown in Table 1. This distribution table is included here to show the distribution of patients in the data set clearly. Of the 545 patients, 198 (36.3%) were female, 257 (47.2%) were above 65 years of age, and 264 (48.4%) were admitted to the ICU. Among the group that included only patients with no NCDs (n=281), 107 (38.1%) were female, and 108 (38.4%) were admitted to the ICU. Moreover, in the group of patients who had one or more NCDs (n=264), 167 (63.3%) were over 65 years of age, and 156 (59.1%) were admitted to the ICU. The age percentile is shown in Figure 2.

**Table 1:** Demographic information for the patients with COVID-19 in each patient group.

Characteristic			Values, n (%)				
		All patients (N=545)		Patients without NCDs^a^ (n=281)		Patients with NCDs (n=264)	
Age >65 years			257 (47.2)		90 (32.0)		167 (63.3)
**Age percentile**							
	10th	115 (21.1)		63 (22.4)		52 (19.7)	
	20th	58 (10.6)		41 (14.6)		17 (6.4)	
	30th	55 (10.1)		38 (13.5)		17 (6.4)	
	40th	60 (11.0)		39 (13.9)		21 (8.0)	
	50th	50 (9.2)		22 (7.8)		28 (10.6)	
	60th	53 (9.7)		24 (8.5)		29 (11.0)	
	70th	55 (10.1)		26 (9.3)		29 (11.0)	
	80th	49 (9.0)		16 (5.7)		33 (12.5)	
	90th	50 (9.2)		12 (4.3)		38 (14.4)	
	>90th	54 (9.9)		15 (5.3)		39 (14.8)	
Female gender			198 (36.3)		107 (38.1)		91 (34.5)
Admitted to ICU^b^			264 (48.4)		108 (38.4)		156 (59.1)

^a^NCDs: noncommunicable diseases.

^b^ICU: intensive care unit.

### Identification of Significant Routine Blood Parameters for SARS-CoV-2 Infection

**Figure 3:**
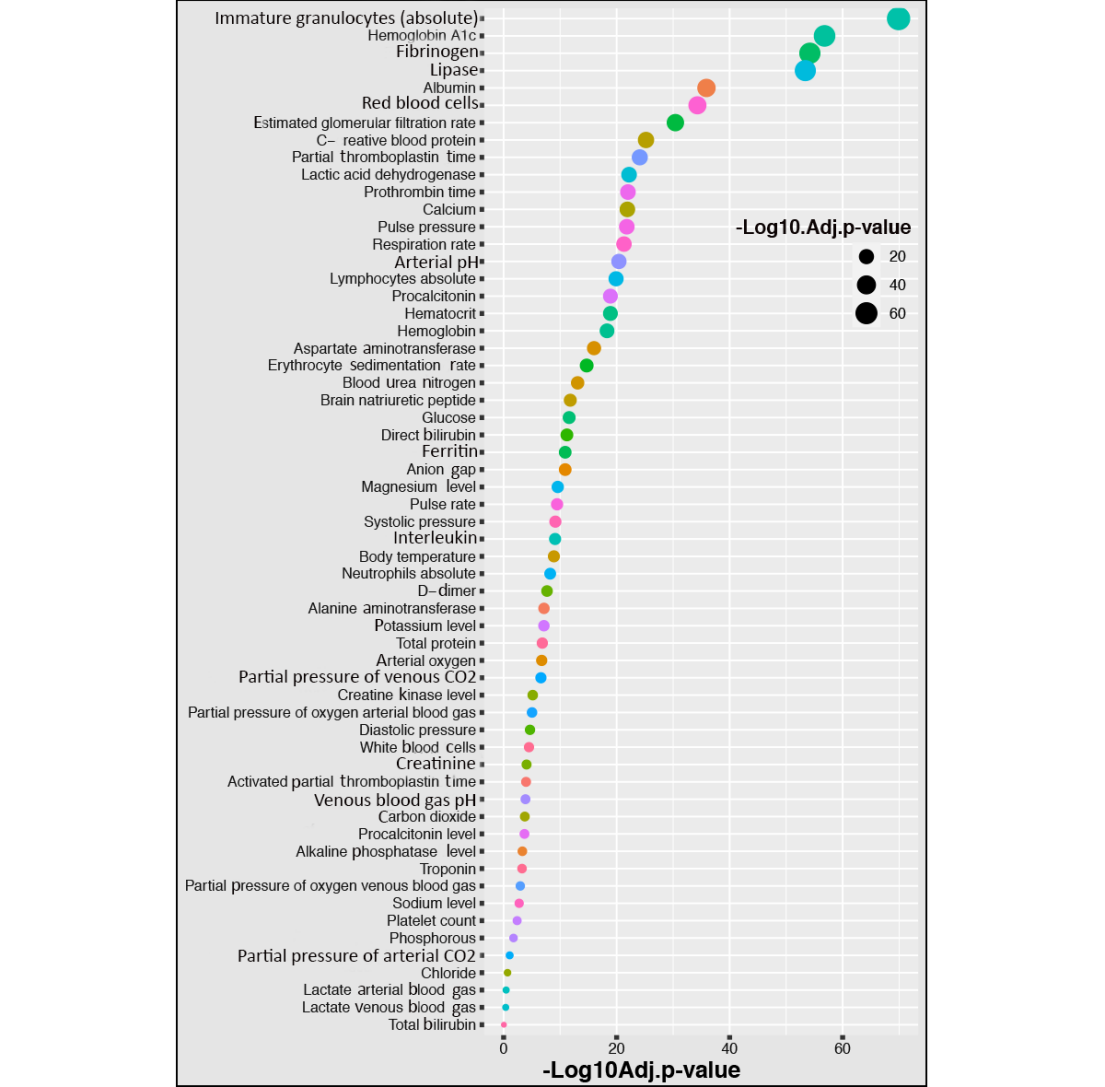
Parameter measurements for various blood parameters and significant differences (using *t* tests) between patients with and without COVID-19. Adj.p-value: adjusted *P* value; D-dimer: dimerized plasmin fragment D.

Our first data set contained 89 blood parameters for confirmed COVID-19–positive patients. Assuming each blood parameter value was normally distributed in the healthy population, we performed Student *t* tests on the tested blood parameters to compare the expected range values (shown in Figure 3) with patients with COVID-19 from the first data set. The combination of Student *t* test and LFC analyses indicated that the 8 most significant candidate predictive parameters for COVID-19 severity status were lipase, C-reactive protein, procalcitonin level, erythrocyte sedimentation rate, brain natriuretic peptide, ferritin, D-dimer, and creatine kinase level, all of which showed *P* values <.001 and absolute LFCs >1.

**Figure 4:**
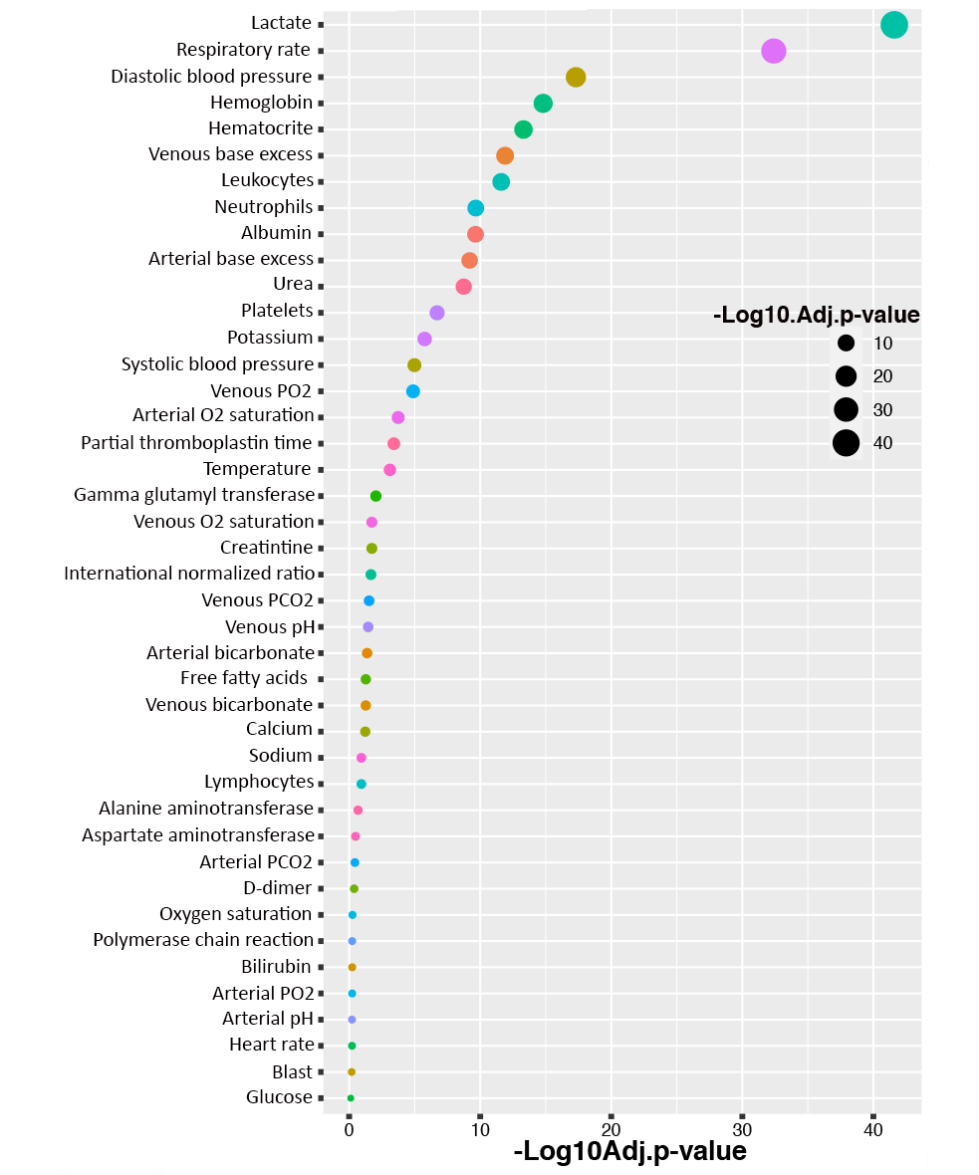
Association of blood parameters with the severity of COVID-19 disease. Associations and significant differences (using *t* tests) between the patients with severe COVID-19 and nonsevere COVID-19. Adj.p-value: adjusted *P* value; D-dimer: dimerized plasmin fragment D; FFA: free fatty acids; GGT: gamma-glutamyl transferase; INR: international normalized ratio.

We applied the Student *t* test to the second data set to attempt to discriminate symptoms of severe and nonsevere COVID-19–positive patients by identifying patient characteristics that are associated with the target variable of disease severity; the analysis results are shown in Figure 4. The most significant blood parameters according to the *t* test results were lactate, respiratory rate, diastolic blood pressure, hemoglobin, hematocrit, venous base excess, leukocytes, neutrophils, albumin, arterial base excess, urea, platelet count, potassium, and systolic blood pressure.

### Clustering and Coexpression Analysis

**Figure 5:**
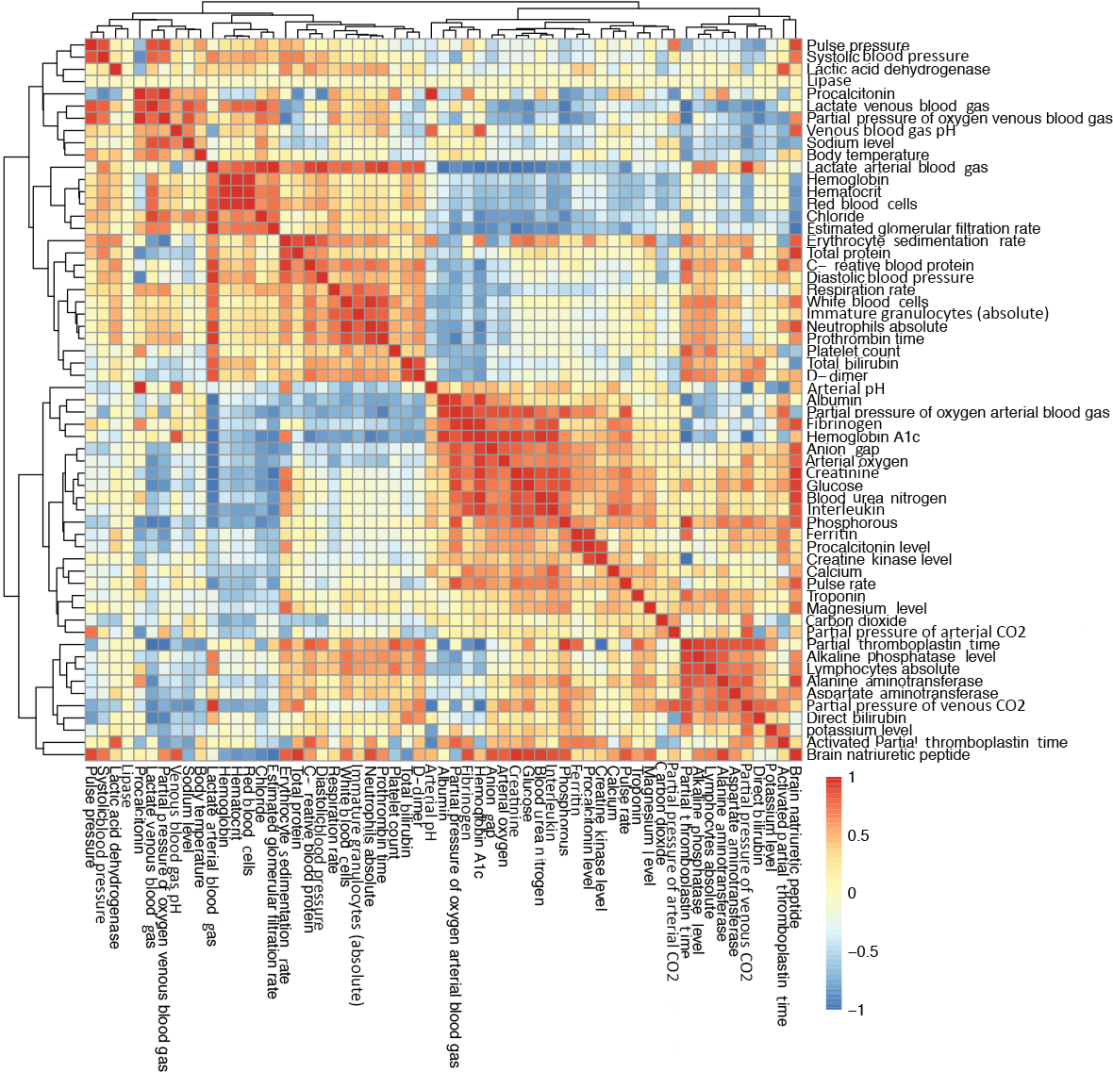
Correlation heat map among the various blood parameters examined using the data set of 89 patients. D-dimer: dimerized plasmin fragment D.

We also performed Pearson correlation tests for the different routine blood parameters. The Pearson correlation results are shown in Figure 5. The purpose of the hierarchical clustering was to observe which blood samples share similar properties in terms of their values among all the patients. We found that some blood features formed clusters, which indicates that they share similar properties among patients. We found that there were indeed some hierarchical clusters in the tests that showed equal significance for all the patients. From the total of 59 blood samples, we found 4 different concordant clusters that were strongly correlated with each other. The first cluster comprised pulse pressure and systolic blood pressure. The second cluster comprised hemoglobin, hematocrit, and red blood cells. The third cluster comprised C-reactive protein, erythrocyte sedimentation rate, diastolic blood pressure, and respiratory rate. Procalcitonin levels, ferritin, and creatine kinase levels composed the fourth cluster.

### Prediction of Severe COVID-19 for Critical Treatment Using ML Models

In this section, we first describe the performance of the various ML models employed and their applications. We then present the most important reduced set of blood and physical sign parameters that can precisely discriminate patients with severe COVID-19 from those with nonsevere disease. The reduced collection of blood parameters is also significant for outcomes of patients with severe COVID-19.

For the ML analysis of the second data set, we applied the respective methods and models; their performances and the evaluation matrices are shown in Table 2. In the data group of all patients with and without NCDs, we found that the RF and GBM methods gave the highest testing accuracy score of 89%, and the other methods and models demonstrated >80% testing accuracy. The highest AUC was obtained for RF and GBM (89%), and other methods and models achieved suitable AUC values >80%. The highest precision value of 91% was observed for XGB and GBM. The highest recall values obtained were 93% for KNN and 90% for RF and LGBM; the other methods showed scores above 80%. The best F1 score was 90% for RF, and the other models showed F1 scores >80%. RF and GBM had the lowest log loss value of 3.8%, and the other methods and models also showed particularly low values (ie, <7%). In this patient group, we saw that all of our applied models achieved good performance in every evaluation matrix with accuracy scores >80%; therefore, in practice, any of the models can be employed.

**Table 2:** Accuracy and evaluation matrices for each data group.

Data set and matrices		RF^a^	LGBM^b^	SVM^c^	DT^d^	XGB^e^	GBM^f^	KNN^g^	ANN^f^
**Combined**									
	Accuracy	0.89	0.88	0.84	0.82	0.88	0.89	0.84	0.83
	AUC^g^	0.89	0.88	0.84	0.82	0.88	0.89	0.84	0.82
	Precision	0.9	0.88	0.84	0.83	0.91	0.91	0.81	0.92
	Recall	0.9	0.9	0.88	0.83	0.86	0.88	0.93	0.69
	F1 score	0.9	0.89	0.86	0.83	0.88	0.89	0.86	0.79
	Log loss	3.8	4.12	5.39	6.34	4.12	3.8	5.39	6.02
**With NCDs^h^**									
	Accuracy	0.91	0.93	0.84	0.84	0.87	0.89	0.77	0.74
	AUC	0.91	0.92	0.83	0.84	0.87	0.89	0.79	0.71
	Precision	0.89	0.89	0.83	0.85	0.82	0.82	0.65	0.77
	Recall	0.97	1	0.91	0.88	0.85	0.9	0.85	0.82
	F1 score	0.93	0.94	0.87	0.86	0.83	0.86	0.74	0.79
	Log loss	3.03	2.42	5.45	5.45	4.56	3.91	7.82	9.12
**Without NCDs**									
	Accuracy	0.93	0.91	0.84	0.86	0.91	0.88	0.74	0.74
	AUC	0.92	0.91	0.83	0.85	0.9	0.86	0.73	0.71
	Precision	0.89	0.91	0.83	0.85	0.89	0.84	0.74	0.86
	Recall	1	0.94	0.91	0.91	0.97	0.97	0.81	0.48
	F1 score	0.94	0.92	0.87	0.88	0.93	0.9	0.78	0.62
	Log loss	2.42	3.02	5.45	4.85	3.03	4.24	9.09	9.09

^a^RF: random forest.

^b^LGBM: light gradient boosting machine.

^c^SVM: support vector machine.

^d^DT: decision tree.

^e^XGB: extreme gradient boosting.

^f^GBM: gradient boosting machine.

^g^KNN: k-nearest neighbor.

^f^ANN: artificial neural network.

^g^AUC: area under the curve.

^h^NCDs: noncommunicable diseases.

In the data group of patients with no NCDs, we found that RF demonstrated the highest accuracy score of 93%, LGBM and XGB performed with 91%, and SVM and DT showed good accuracy scores of >80%. However, KNN and ANN showed comparatively low accuracy scores of 74% because when we divided the data set, the size of the data was small. RF demonstrated the highest AUC of 92%; the AUC of LGBM was 91% and that of XGB was 90%. LGBM showed the highest precision value of 91%, while RF and XGB showed values of 89%. The highest precision value was 91% for LGBM, and other methods and models had values >80% except for KNN (74%). The highest recall values were 100% for RF and 97% for XGB and GBM; the other methods and models showed values above 80%, except ANN (48%). RF achieved the highest F1 score of 94%; XGB achieved a score of 93%, LGBM scored 92%, and SVM and DT scored 88%. However, KNN and ANN achieved comparatively low F1 scores, with 78% and 62% respectively, because of the lower training sample sizes. The lowest log loss value was 2.42% for RF, and the other methods and models also demonstrated good log loss values below 10%. In this patient group, we observed that excepting KNN and ANN, all of the models achieved accuracy scores >80%, and the evaluation matrix showed good model performance. Therefore, the best-performing models could be usefully applied in clinical scenarios.

In the data group of patients who had one or more coexisting NCDs, we found that LGBM performed with the highest accuracy score of 93%, and RF, GBM, XGB, SVM, and DT achieved scores of 91%, 89%, 87%, 84%, and 84%, respectively. KNN and ANN performed poorly, showing 77% and 74% accuracy, respectively; however, this result was due to the small amount of available data. The highest AUC score was 92% for LGBM, and RF, SVM, DT, XGB, GBM, KNN, and ANN scored 91%, 83%, 84%, 87%, 89%, 79% and 71%, respectively. RF and LGBM demonstrated the highest precision value of 89%, and the other methods and models performed with good precision values >80%, except for KNN and ANN. LGBM achieved the highest recall value of 100%, RF achieved 97%, GBM 90%, SVM 83%, and DT 88%; the other methods and models performed above 80%. The highest F1 score was 94% for LGBM; RF also demonstrated 93%, and the other methods and models performed above 80% except for KNN and ANN. KNN and ANN achieved F1 scores of 74% and 79%, respectively; however, the number of training samples for these models was small.

**Figure 6:**
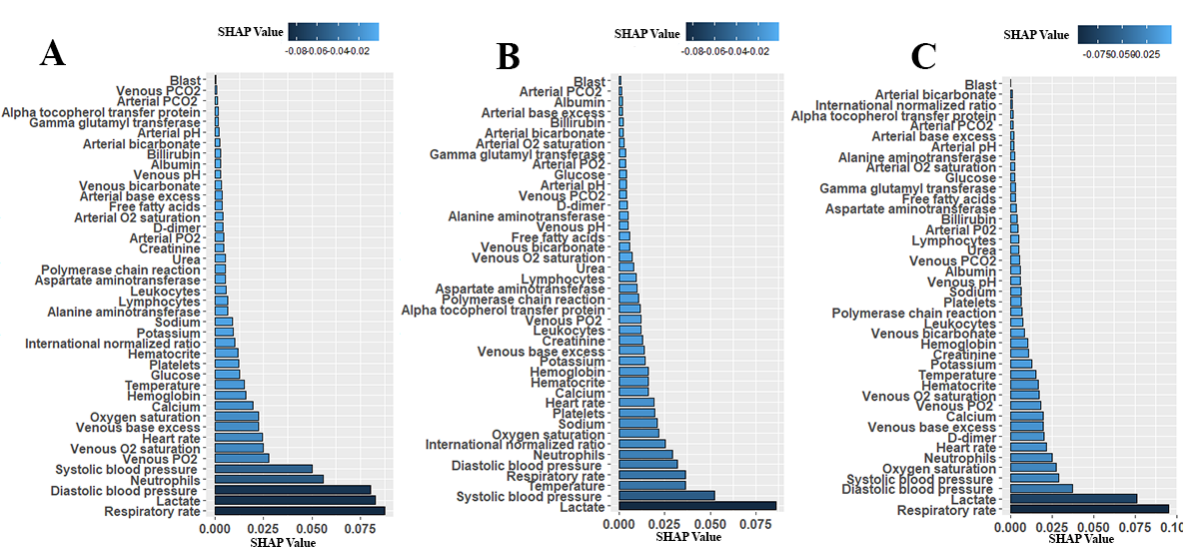
Sorted significant and impacted blood parameters of patients with COVID-19 based on SHAP values, defined as the coefficient values of each parameter after model training: (A) combined patients group; (B) patients with noncommunicable diseases; (C) patients without noncommunicable diseases. Artificial intelligence models were used to identify the most predictive blood parameters for the severity of COVID-19 symptoms. Higher coefficient values of machine learning model outcomes indicate a higher significant association with disease severity. D-dimer: dimerized plasmin fragment D; FFA: free fatty acids; GGT: gamma-glutamyl transferase; INR: international normalized ratio; SHAP: Shapley Additive Explanations; TTPA: partial thromboplastin time.

Using ML analysis, we attempted to determine the most significant blood parameters that are highly predictive for identifying patients with severe COVID-19. We found the SHAP (Shapley Additive Explanations) values for each of the ML algorithms, quantile-normalized those values, and finally calculated the average values for each blood parameter. In Figure 6, the parameter list sorted according to the feature importance level (average SHAP value) is presented. In this figure, the left panel shows the combined patients (those with NCDs and those without NCDs), the middle panel shows the patients who have NCDs only, and the right panel shows the patients who have no NCDs.

In the above analysis, we observed that a small set of blood parameters had high SHAP values, which indicates that those parameters are impactful and predictable for the diagnosis of severe COVID-19. According to the level of importance, respiratory rate, lactate, blood pressure (diastolic and systolic), neutrophils, and oxygen saturation level were the most significant and common parameters for the group including all the patients. The exceptional cases are venous PO_2_, venous saturated O_2_, and heart rate, which were impactful for the combined patient group, and temperature and INR, which were impactful for the group of patients with NCDs only.

In the statistical analysis, it was found that the absolute value of lymphocytes is a key predictor for severe patient outcomes. The value of the lymphocytes parameter decreased with increasing severity level of the patients with COVID-19. We also observed the opposite scenario for neutrophil data, as in, the lymphocytes parameter increased if the patient’s condition deteriorated toward a severe situation.

## Discussion

### Principal Findings

During the worldwide outbreak of COVID-19, classifications of disease mortality risk are of very great significance in prevention and treatment allocation. In this investigation, we identified a number of blood analysis parameters that can be used as risk factors for the assessment of disease severity in patients with COVID-19. We developed predictive algorithms that use a large number of blood parameters and demonstrated that these methods have potential to predict the disease severity of patients with COVID-19 with high accuracy.

We identified a number of features of patient data that contributed strongly to the predicted value of the algorithms (ie, were found to contribute to the accuracy of all our best ML algorithms), some of which were not obvious candidate predictors. We found that the absolute value of lymphocytes in the group of patients with severe symptoms was consistently lower than that in the nonsevere symptom group. The neutrophil parameters of the severe symptom group were higher than those of the nonsevere symptom group. A high neutrophil level indicates a heightened level of immune activation and may play a role in the “inflammatory storm” that is characteristic of severe COVID-19 symptoms, which results in great harm to tissues and cells [28]. Low lymphocyte levels may reflect impeded antibody-based immune cell functions, which are suspected to result in patients with severe COVID-19 who are susceptible to bacterial infection [29]. Our results suggest that the numbers of circulating lymphocytes in the patients who developed severe symptoms were significantly lower than those in patients who did not have severe symptoms. In contrast, the inclusion of neutrophils in the severe patients in the ICU showed a greater influence, which is consistent with the findings of Qin et al [30].

We found that the indicator factors could be reliable predictors that discriminated between patients with severe and nonsevere COVID-19. Recent work has revealed the utility of routine blood parameters in the screening of patients with COVID-19. This is facilitated by the fact that blood parameter analysis is generally fast, affordable, and promptly accessible in the same health facility where patients are receiving treatment. The pathological tests of patients with COVID-19 identified abnormalities in some blood parameters. In previous published studies, a number of altered blood parameters in patients with COVID-19 who developed severe symptoms were identified in addition to the lymphocyte and neutrophil parameters noted above, such as eosinophils, basophils, monocytes, platelets, and total leukocytes as well as serum levels of urea, potassium, hemoglobin, and C-reactive blood protein [31-33]; this provides supportive evidence for our findings. Li et al [34] identified that bacterial infection affected COVID-19 pneumonia in some cases of mortality. Bacterial contamination also causes expanded leucocyte count and neutrophil count, which may be linked to defective immune responses. A few patients with COVID-19 have abnormal blood coagulation function: prothrombin time and D-dimer level increase [28], while thrombosis is linked with expanded platelet consumption and diminished platelet number.

Respiratory rate is one of the principal vital signs for symptom severity in patients with COVID-19. Abnormally high respiratory rates (<12 or >25 breaths/min) are also seen in a range of conditions, including asthma, heightened anxiety, pneumonia, congestive heart failure, and lung disease (all of which exacerbate COVID-19 conditions when presenting as comorbidities) and are a significant feature in severely affected patients with COVID-19 [35,36]. Elevated heart rate is similarly a key sign [37] and may be a cause of dizziness or shortness of breath in patients with sCOVID-19 [38]. Blood pressure is additionally a clinical sign for patients with COVID-19 [39]. Hypoxemia is also a sign that indicates a below-average level of oxygen saturation in the blood. The usual range of arterial oxygen is approximately 75-100 mm Hg, and a pulse oximeter reads the expected range from 95% to 100%; below 90% indicates that the patient’s condition is critical [40]. This finding is often observed in patients with COVID-19 who may lack other obvious symptoms; therefore, it is a particularly dangerous feature of the disease. The serum lactic acid test is also a significant test that indicates disease severity in patients with COVID-19. Typically, the level of lactate in the blood is very low; a rise in lactate level is typically associated with low oxygen levels [41,42].

In summary, a number of signs and symptoms can indicate that COVID-19 is likely to become severe in a patient. A standardized and objective way to combine these and other less obvious predictors in a way that can optimize patient outcomes and resource management is needed. Our methodology, described here and derived from a number of different ML algorithms, can provide such an improved method. Indeed, the fact that high accuracy was obtained using similar predictors by different ML algorithms (indicating that there is limited sensitivity to the methodology) can provide confidence that these parameters are useful and that the approach is a sound one.

### Conclusion

The results of our analysis indicated that there is a strong relationship between particular abnormal blood parameters and disease severity status in hospitalized patients with COVID-19. The primary utility of our findings is that the subset of routine blood parameters linked to disease severity could be used in a predictive algorithm that would better enable appropriate care to be given before the onset of severe symptoms. This is of particular importance in developing countries, where ICU beds in hospitals are a limited resource. This can be achieved using a relatively small number of currently available blood-based hospital tests to properly use ICU resources and identify patients who need to be monitored closely.

Among the association between blood parameters that can give predictive information regarding the severity of COVID-19 symptoms, the levels of lactate and immature granulocytes (absolute) appeared to have the strongest predictive value. Levels of hemoglobin, procalcitonin, erythrocyte sedimentation rate, brain natriuretic peptide, ferritin, D-dimer, and platelets likewise showed significant deviation from the normal control group for prediction of disease severity. Other parameters, namely respiratory rate, lactate, blood pressure (systolic and diastolic), hematocrit, venous and arterial base excess, neutrophils, albumin, and urea, showed less obvious deviations but clearly had predictive value. Our work suggests that links exist between these parameters and COVID-19, and similar proinflammatory infectious diseases may merit more detailed physiological investigations.

There were a few limitations to our study. First, the small sample size may restrict the precision of the identification of severity. Second, the absence of more detailed clinical information in the data sets that were used (such as patient age, sex, and comorbidities) may hinder better classification, although this suggests that in future studies, we could use new data sets to address this and improve on our work. Finally, the disease severity and mortality of COVID-19 varies significantly from country to country; the reasons for this are very poorly understood, but it is suggested that this type of predictive analysis should be conducted on data from other parts of the world to improve the performance of the algorithm. Nevertheless, we hope our study can be used by practitioners and help policy makers to improve resource allocation and outcomes for patients with COVID-19.

## Abbreviations

ANN: artificial neural network
AUC-ROC: area under the receiver operator characteristic curve
D-dimer: dimerized plasmin fragment D
DT: decision tree
GBM: gradient boosting machine
ML: machine learning
NCD: noncommunicable disease
ICU: intensive care unit
INR: international normalized ratio
KNN: k-nearest neighbor
LFC: log 2 fold change
LGBM: light gradient boosting machine
RF: random forest
RT-PCR: reverse transcription–polymerase chain reaction
SHAP: Shapley Additive Explanation
SVM: support vector machine
XGBoost: extreme gradient boosting
